# Improving Vitamin D Status in Preterm Newborns: A Randomized Trial of 800 vs. 400 IU/Day

**DOI:** 10.3390/nu17111888

**Published:** 2025-05-30

**Authors:** Nawinda Rueang-amnat, Kulnipa Kittisakmontri, Varangthip Khuwuthyakorn, Shanika Kosarat, Satit Manopunya, Mallika Pomrop

**Affiliations:** 1 Division of Neonatology, Department of Pediatrics, Faculty of Medicine, Chiang Mai University, Chiang Mai 50200, Thailand; 2 Division of Pediatric Nutrition, Department of Pediatrics, Faculty of Medicine, Chiang Mai University, Chiang Mai 50200, Thailand

**Keywords:** preterm newborns, hypovitaminosis D, vitamin D3, vitamin D supplementation

## Abstract

**Background and Aims:** Preterm newborns are particularly susceptible to hypovitaminosis D, potentially impairing bone mineralization. In Thailand, data on its prevalence and standardized supplementation protocols remain limited. This study aimed to compare the efficacy of two vitamin D3 dosages (400 IU/day vs. 800 IU/day) in improving serum vitamin D concentrations and metabolic bone parameters in preterm newborns. **Methods**: A randomized controlled trial was conducted in preterm newborns born at ≤32 weeks’ gestation or with birth weight ≤1500 g. Preterm newborns were randomized to receive either 400 IU or 800 IU/day of vitamin D3. Serum 25-hydroxyvitamin D (25(OH)D) was measured using electrochemiluminescence immunoassay (ECLIA). Metabolic bone parameters—including calcium, phosphorus, alkaline phosphatase, and albumin—were assessed at baseline and again at six weeks of age. **Results:** Of the 38 enrolled infants, baseline 25(OH)D levels were comparable between groups (14.8 ± 4.8 ng/mL in the 800 IU/day group vs. 14.7 ± 6.9 ng/mL in the 400 IU/day group). At six weeks, the 800 IU group demonstrated significantly higher 25(OH)D levels (47.3 ± 21.0 ng/mL vs. 32.0 ± 14.2 ng/mL; *p* = 0.013), with a large effect size (Cohen’s d = 0.85) and the difference-in-differences of +15.7 ng/mL. The prevalence of hypovitaminosis D declined from 89% to 5% in the 800 IU/day group and from 74% to 32% in the 400 IU/day group (*p* = 0.036). No significant differences in metabolic bone parameters or signs of toxicity were observed. **Conclusions:** Vitamin D3 supplementation at 800 IU/day significantly improved vitamin D status and reduced hypovitaminosis D in preterm newborns, without observed toxicity.

## 1. Introduction

Vitamin D is a fat-soluble vitamin that plays an essential role in maintaining calcium and phosphate homeostasis by enhancing intestinal absorption and promoting their renal reabsorption. It is critical for promoting bone mineralization and skeletal growth, particularly during periods of rapid growth such as infancy and early childhood [1,2]. In addition to its skeletal functions, vitamin D contributes to various physiological processes, including modulation of innate and adaptive immune responses, regulation of cellular growth and differentiation, and maintenance of cardiovascular and metabolic function [1,2,3,4,5]. In preterm newborns, vitamin D deficiency is a common and often asymptomatic condition. However, if left undetected and untreated, it can lead to serious complications such as rickets.

Preterm newborns are at increased risk of vitamin D deficiency due to insufficient time for in utero accumulation of vitamin D, calcium, and phosphate, particularly during the third trimester, a critical period for fetal bone mineralization. After birth, exclusive breastfeeding and insufficient sunlight exposure are additional risk factors for vitamin D deficiency [3,5,6,7,8,9,10]. The daily vitamin D requirement of preterm newborns often exceeds the level provided by breast milk and formula. While breast milk contains only 15–50 IU/L of vitamin D, infant formula provides approximately 400 IU/L [4,5].

Vitamin D deficiency has been recognized as a global health concern. In Asia, the prevalence of deficiency ranges from 45 to 98% in the general population and 78–98% among pregnant women [8]. In Thailand, studies report a prevalence of vitamin D deficiency among pregnant women ranging from 22 to 34% [11,12,13], which may lead to a deficiency in their newborns [3,9].

Studies in Thailand have shown that 57% of full-term infants aged 0–6 months, 28.3% of those aged 6–12 months, and 24.5–31.3% of children aged 3–6 years are vitamin D deficient [7,14,15]. However, data on the incidence of vitamin D deficiency in preterm newborns in Thailand remain limited.

Vitamin D deficiency can significantly impact bone health, growth, and organ function in newborns. Thus, ensuring adequate vitamin D intake is essential. International health organizations have issued recommendations for vitamin D intake across age groups, particularly preterm newborns. The American Academy of Pediatrics (AAP) recommended 200–400 IU/day of vitamin D for newborns weighing less than 2500 g [2], whereas the European Society for Paediatric Gastroenterology Hepatology and Nutrition (ESPGHAN) 2022 guidelines recommended 400–700 IU/kg/day, with a maximum of 1000 IU/day [16].

Serum vitamin D levels can be influenced by geographical location, sunlight exposure, and dietary intake [10,17]. International studies have explored optimal vitamin D supplementation strategies in both term and preterm newborns [3,10,18,19,20,21,22,23]. In Thailand, the Thai Pediatric and Adolescent Endocrine Society currently recommends 400 IU/day of supplemental vitamin D for full-term newborns. However, there are no national guidelines specifically tailored for preterm newborns. Furthermore, limited research exists on optimal dosing or the clinical effects of vitamin D supplementation in Thai preterm populations.

Given the lack of data on the prevalence of hypovitaminosis D and its clinical impact in preterm newborns in Thailand, further research is needed to inform evidence-based supplementation strategies. Such strategies are essential to prevent hypovitaminosis D and its complications, and to support the development of appropriate screening and supplementation guidelines for Thai preterm newborns. Therefore, this study aimed to compare serum vitamin D levels and metabolic bone parameters in preterm newborns receiving 400 IU/day vs. 800 IU/day of vitamin D, and to assess the prevalence of hypovitaminosis D in this population.

## 2. Methods

### 2.1. Study Design

This was a single-center, randomized controlled trial conducted in the Neonatal Intensive Care Unit (NICU) of Maharaj Nakorn Chiang Mai Hospital, Thailand, from March 2024 to February 2025. The study received ethical approval from the Research Ethics Committee of the Faculty of Medicine, Chiang Mai University (Study code: PED-2566-0538) and was registered with the Thai Clinical Trials Registry (TCTR) under registration number TCTR20240322002. Written informed consent was obtained from the parents or legal guardians of all participants prior to enrollment. Participation was voluntary, and standard care was provided regardless of enrollment. Families retained the right to decline or withdraw consent at any time without consequences.

### 2.2. Participants

Eligible participants were (1) preterm newborns with a gestational age ≤ 32 weeks or a birth weight of ≤1500 g; (2) born at Maharaj Nakorn Chiang Mai Hospital or referred from other hospitals within 48 h of birth; (3) managed in accordance with the hospital’s nutritional guidelines for critically ill newborns; and (4) able to tolerate enteral feeding at ≥120 mL/kg/day within 2 weeks after birth.

Exclusion criteria included chromosomal abnormalities, critical congenital heart disease, congenital gastrointestinal anomalies requiring surgery (e.g., gastroschisis, omphalocele, or intestinal atresia), multiple anomalies, diagnosed with definite necrotizing enterocolitis (modified Bell’s criteria stage ≥ 2), and diagnosed with inborn errors of metabolism. Newborns whose parents declined or withdrew consent were also excluded.

Withdrawal criteria included newborns who died before the 6-week blood sampling, interruption of enteral feeding ≥7 days after reaching full feeding, biliary abnormalities such as cholestasis or clinical signs suggestive of vitamin D toxicity (e.g., frequent unexplained vomiting, seizures, or arrhythmia).

### 2.3. Randomization

Participants were randomized in a 1:1 ratio using randomization with a fixed block size of four. Group assignments were prepared in advance, sealed in opaque, sequentially numbered envelopes, and opened only after confirming eligibility and obtaining informed consent. The allocation sequence was generated by an independent investigator to ensure allocation concealment.

### 2.4. Intervention

After enrollment, newborns were randomly assigned to receive either 400 IU/day or 800 IU/day of vitamin D3 supplementation. Both groups received standard multivitamin drops (B.L.HUA, Bangkok, Thailand) 0.5 mL/day, containing 200 IU of vitamin D3. To meet the targeted dosage, an additional 200 IU/day or 600 IU/day of vitamin D3 (B.L.HUA, Bangkok, Thailand) was administered for the 400 IU/day and 800 IU/day groups, respectively. Supplementation began once the infant tolerated oral feeding ≥120 mL/kg/day. Vitamin D3 was used exclusively in this study, as it has been shown to be more effective than vitamin D2, which is generally recommended for supplementation [24].

NICU staff were not blinded to group assignment due to the nature of the intervention; however, laboratory personnel responsible for biochemical analyses were blinded to treatment allocation.

Newborns received exclusive breast milk, pasteurized donor human milk (PDHM), formula, or a combination thereof, depending on maternal milk availability. Human milk fortifiers (HMFs) or preterm formula were added when enteral feeding reached ≥80 mL/kg/day, with HMFs preferred when available. Additional vitamins and minerals were supplemented according to standardized protocols. The hospital’s criteria for PDHM usage, transition to formula, and fortification strategy are detailed in Supplementary Materials.

### 2.5. Laboratory Measurements and Definitions

Serum 25(OH)D level and metabolic bone laboratory parameters, including serum alkaline phosphatase, serum calcium, serum phosphorus, and serum albumin [25,26] were measured prior to initiating vitamin D supplementation and again at six weeks of age, in accordance with the guidelines of the AAP [2] to evaluate the impact of the supplementation. Vitamin D status was classified based on national guidelines as follows: deficiency (<12 ng/mL), insufficiency (≥12 to <20 ng/mL), sufficiency (≥20 to 100 ng/mL), and toxicity (>100 ng/mL) in the presence of clinical signs such as hypercalcemia, hypercalciuria, or suppressed parathyroid hormone (PTH) levels [27].

Serum 25(OH)D concentrations were measured using an electrochemiluminescence binding assay on the Cobas e 411 analyzer (Roche Diagnostics, Rotkreuz, Switzerland), with a detection range of 3.00–120 ng/mL and a coefficient of variation (CV) of ≤20%. Serum calcium was measured photometrically using the Cobas c 303 analyzers (Roche Diagnostics), with a detection range of 0.8–20.1 mg/dL and CV ≤30%. Serum phosphorus was measured using the molybdate UV method on the same analyzer, with a detection range of 0.31–20.0 mg/dL and a CV of ≤30%. Serum alkaline phosphatase was measured using a colorimetric method on the Cobas c 303 analyzer, with a detection range of 5–1200 U/L and a CV of ≤20%.

### 2.6. Safety and Monitoring

At six weeks of age, blood tests were evaluated for abnormalities suggestive of metabolic bone disease in preterm newborns. These included low serum phosphorus (<5.5 mg/dL), elevated serum alkaline phosphatase (>450 U/L), low corrected serum calcium (<8.5 mg/dL), and low 25(OH)D levels (<20 ng/mL). Supplementation was adjusted accordingly based on the results. In cases of vitamin D deficiency, the dosage was increased by 200 IU/day above the assigned dose. If vitamin D toxicity was suspected, supplementation was discontinued. Serum 25(OH)D levels were re-evaluated four weeks after adjustment. A pediatric endocrinologist was consulted in all cases of abnormal findings or suspected toxicity.

Routine laboratory monitoring at our institution included blood testing when the newborn reached an enteral feeding volume of ≥120 mL/kg/day, and again at 2, 4, and 6 weeks postnatal age. Monitoring continued every 4 weeks until the newborns reached 40 weeks postmenstrual age or were discharged. Serum calcium was evaluated at each time point. If hypercalcemia (>11 mg/dL) was detected before 6 weeks of age, which may indicate vitamin D toxicity, an early 25(OH)D level was obtained prior to the scheduled six-week testing.

### 2.7. Sample Size

Sample size was calculated using the non-parametric Mann–Whitney U test with a two-sided significance level (α) of 0.05 and 80% power. The calculation was based on the observed difference in median serum 25(OH)D levels between preterm newborns receiving 400 IU/day and 800 IU/day of vitamin D supplementation (33.9 vs. 42.2 ng/mL), as reported by Anderson-Berry et al. [3] and the estimated probability of superiority was approximately 0.80. Under these assumptions, the required sample size was calculated to be 15 newborns per group. Allowing for an anticipated 25% dropout rate, the final estimated sample size was 19 newborns per group.

### 2.8. Statistical Analysis

Statistical analyses were conducted using SPSS for Windows version 26 (IBM Corp., Armonk, NY, USA) based on the intention-to-treat (ITT) principle. Statistical significance was set at a *p*-value of < 0.05. Normality of data distribution was assessed using the Shapiro–Wilk test. Continuous variables with a normal distribution were analyzed using independent *t*-tests for between-group comparisons and paired *t*-tests for within-group comparisons, while non-normally distributed variables were analyzed using the Mann–Whitney U test. Between-group differences in changes from baseline were evaluated using both independent *t*-tests and a difference-in-differences (DiD) analysis to estimate treatment effects, adjusting for time and baseline variation. To evaluate clinical relevance, the effect size (Cohen’s d), absolute risk reduction (ARR), and number needed to treat (NNT) to prevent hypovitaminosis D were calculated, along with their corresponding 95% confidence intervals (CIs).

## 3. Results

**Figure 1:**
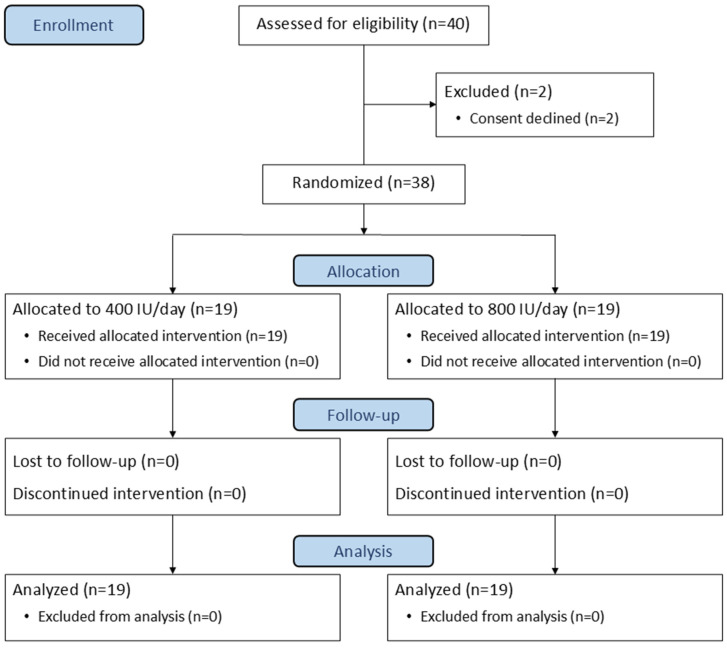
CONSORT diagram of participant flow.

A total of 40 preterm infants were assessed for eligibility between March 2024 and February 2025. Two were excluded prior to intervention (Figure 1), leaving 38 newborns (19 per group) who completed the study and were included in the final analysis. Baseline characteristics were compared between groups, except for a significantly higher proportion of male newborns in the 800 IU group (*p =* 0.003), as shown in Table 1.

At baseline, 82% of all neonates had hypovitaminosis D, with a prevalence of 74% in the 400 IU/day group and 89% in the 800 IU/day group (*p* = 0.209). The mean serum 25(OH)D levels in the 400 IU/day and 800 IU/day groups were 14.7 ± 6.9 ng/mL and 14.8 ± 4.8 ng/mL (*p* = 0.993), respectively. Baseline metabolic bone parameters did not differ significantly between the two groups (Table 2).

After six weeks of supplementation, both groups demonstrated significant within-group increases in serum 25(OH)D levels (*p* < 0.001 for both). The 800 IU/day group showed significantly higher mean 25(OH)D concentrations than the 400 IU/day group (47.3 ± 21.0 vs. 32.0 ± 14.2 ng/mL; *p* = 0.013). The prevalence of hypovitaminosis D was significantly lower in the 800 IU/day group than in the 400 IU group (5% vs. 32%; *p* = 0.036), and no cases of vitamin D deficiency were observed in the 800 IU group.

Most newborns were fed breast milk or pasteurized donor human milk fortified with preterm formula throughout the study period (Table 1). Subgroup analysis of feeding practices showed no significant difference in post-supplementation 25(OH)D levels between infants fed breast milk fortified with preterm formula versus those fortified with human milk fortifier, in both the 800 IU (46.6 ± 19.4 vs. 50.9 ± 33.3 ng/mL; *p* = 0.754) and 400 IU (31.3 ± 13.7 vs. 38.6 ± 22.7 ng/mL; *p* = 0.504) groups.

**Figure 2:**
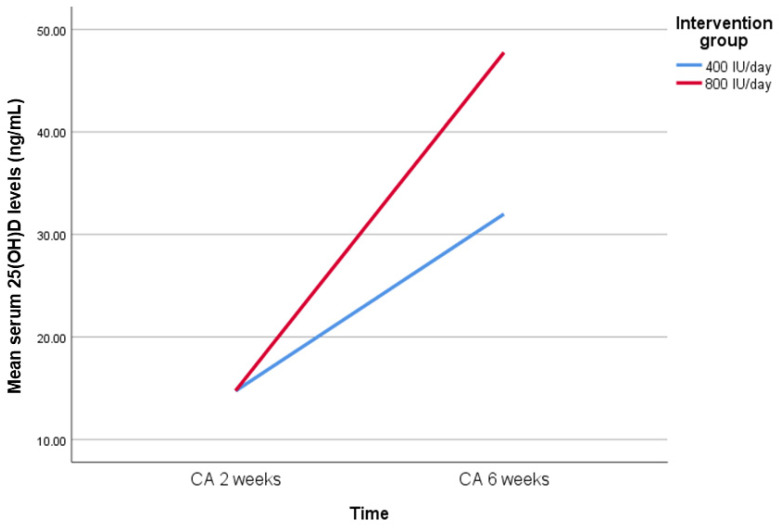
Serum 25(OH)D levels at baseline and after six weeks of supplementation with either 400 IU/day or 800 IU/day of vitamin D3. Data are presented as mean values. The difference-in-differences (DiD) estimate between groups was 15.7 ng/mL (95% CI: 3.4 to 28.1, *p* = 0.013). Abbreviation: 25(OH)D, 25-hydroxyvitamin D; CA, chronological age.

To evaluate clinical relevance, the effect size (Cohen’s d) was 0.85 (95% CI: 0.19 to 1.51). The difference-in-differences (DiD) estimate for serum 25(OH)D levels between the two groups was 15.7 ng/mL (95% CI: 3.4 to 28.1, *p* = 0.013), as shown in Figure 2. The ARR was 27% (95% CI: 3.2% to 49.4%), corresponding to a number needed to treat (NNT) of four (95% CI: 2 to 31) to prevent one case of hypovitaminosis D.

There were no significant differences between the groups in mean metabolic bone parameter levels, nor were there notable changes over the six-week period, except for a slight increase in serum phosphorus levels (Table 2). No cases of hypervitaminosis D were reported in either group.

## 4. Discussion

This randomized controlled trial demonstrated that supplementation with 800 IU/day of vitamin D3 resulted in a significantly greater serum 25(OH)D concentration compared to the 400 IU/day group among preterm neonates born at ≤32 weeks’ gestation or birth weight of ≤1500 g. These findings are consistent with several previous studies from various regions that evaluated different doses of vitamin D3 supplementation in preterm newborns [3,10,22]. These comparisons support the dose-dependent effect of vitamin D3 on serum levels across diverse populations and underscore the potential influence of geographic, maternal dietary, and genetic factors on baseline vitamin D status and supplementation response. The observed efficacy of the higher dose is supported by biological mechanisms, as preterm neonates have a lower cutaneous synthesis capacity due to minimal sun exposure and immature skin [28], lower maternal–fetal transfer during the third trimester, and reduced vitamin D stores at birth [29]. Additionally, impaired intestinal absorption and immature hepatic 25-hydroxylation pathways may further contribute to the deficiency [30]. These physiological limitations underscore the rationale for higher initial dosing strategies to achieve sufficiency in this high-risk neonatal population.

The prevalence of hypovitaminosis D in our study was 82% at baseline and aligned with previous studies, where prevalence ranged between 72 and 80.2% [10,18,22]. At six weeks, the prevalence of hypovitaminosis D was markedly reduced in the 800 IU/day group, with only 5% remaining insufficient, and no cases of deficiency were observed. These findings are consistent with previous studies reporting improved vitamin D status following higher-dose supplementation [10,21]. This improved vitamin D status may contribute to better bone health and reduced long-term risks of metabolic bone disease in preterm newborns, although long-term outcomes were not evaluated in this study.

A large between-group effect size and significant findings from the DiD analysis confirmed that the 800 IU/day dose was more effective in improving serum 25(OH)D levels than the lower dose. Furthermore, the reduction in hypovitaminosis D was clinically meaningful, with a clinically relevant ARR and a low NNT, suggesting that only a few infants require higher-dose supplementation to prevent insufficiency in one case. These findings support the rationale for adopting higher-dose regimens to more effectively correct deficiency in this high-risk population. However, the relatively wide confidence intervals observed in this study suggest some imprecision in effect estimates, likely due to the limited sample size.

The 800 IU/day dose was well tolerated, with no observed cases of vitamin D toxicity, consistent with previous reports [3]. Although some studies have reported occasional elevated 25(OH)D levels at similar doses [10,21], no such findings occurred in our study. The relatively low vitamin D content of breast milk compared to preterm formula [27] may have contributed to this favorable safety profile. However, in settings where formula is the primary source of nutrition, cumulative intake should be closely monitored to avoid potential overdose. The influence of feeding practices on vitamin D status was also explored in this study. Throughout the study, most newborns were fed breast milk or pasteurized donor milk fortified with preterm formula rather than human milk fortifier. Despite differences in vitamin D content between the two types of fortification, as shown in the supplementation details, no significant differences in post-supplementation 25(OH)D levels were observed between fortification types.

There was no significant difference in metabolic bone laboratory parameters or short-term clinical outcomes between the two groups, both at baseline and after six weeks of supplementation. These findings are consistent with previous studies [3,10,22]. As observed in our study, serum 25(OH)D levels changed earlier than metabolic bone laboratory parameters. The inclusion of specific bone turnover markers such as bone-specific alkaline phosphatase (BALP), osteocalcin, procollagen type I *N*-terminal propeptide (P1NP), and *C*-terminal telopeptide of type I collagen (CTX) could have enhanced the assessment of skeletal response [31]. However, these markers are not routinely used in neonatal practice due to the lack of standardized reference ranges for preterm infants and the high biological variability in this age group [32]. Therefore, routine monitoring of 25(OH)D levels may provide an earlier and more reliable indication of vitamin D deficiency, guiding individualized dosing adjustments.

Maternal vitamin D status plays a key role in determining neonatal 25(OH)D levels, particularly in preterm infants. In Thailand, routine vitamin D supplementation during pregnancy is not part of standard care, and previous studies report deficiency rates of 22–34% among pregnant women [11,12,13]. Although maternal levels were not measured in our study, suboptimal maternal status may have contributed to the high prevalence of neonatal hypovitaminosis D. Future studies should consider incorporating maternal vitamin D assessment to better understand the maternal–fetal transfer and its impact on neonatal outcomes.

This study has several limitations. First, the relatively small sample size may limit the generalizability of the findings and contribute to wide confidence intervals for some estimates, such as the ARR and NNT. Second, although randomization was performed appropriately, a significantly higher proportion of male newborns was observed in the 800 IU/day group. While sex has not been consistently shown to affect serum vitamin D levels in preterm newborns, this imbalance could introduce a potential source of confounding. However, current evidence suggests that maternal vitamin D status is a more significant determinant of neonatal vitamin D levels than newborn sex [33]. Third, although clinical staff were not blinded to group allocation, standardized feeding and fortification protocols minimized potential bias. Importantly, laboratory personnel were blinded to treatment groups, reducing detection bias. Finally, the study was conducted at a single center, which may limit its external validity.

Although this study confirmed that 800 IU/day of vitamin D supplementation improved vitamin D status in preterm newborns, its long-term benefits on growth and bone outcomes remain to be determined. Future multicenter trials, larger sample sizes, stratified samples, and extended follow-up are recommended.

## 5. Conclusions

In preterm newborns with a GA of ≤32 weeks or BW ≤ 1500 g, supplementation with 800 IU/day of vitamin D3 effectively increases 25(OH)D levels and reduces hypovitaminosis D prevalence at six weeks of age, without causing toxicity in predominantly breast milk-fed newborns. Although no significant changes in metabolic bone parameters were observed, early monitoring of vitamin D status remains important to guide individualized supplementation strategies and prevent long-term complications.

## Figures and Tables

**Table 1:** Baseline characteristics.

Characteristics	800 IU (n = 19)	400 IU (n = 19)	*p*-Value
Male sex, n (%)	16 (84)	7 (37)	0.003 *
Gestational age, week, mean ± SD	30.5 ± 2.3	31.1 ± 2.4	0.425
Birth weight, grams, mean ± SD	1169 ± 370	1349 ± 255	0.091
Small for gestational age, n (%)	6 (32)	2 (11)	0.111
APGAR score at 5 min, median (range)	8 (6–10)	8 (4–10)	0.674
Duration of PN day, median (range)	8 (6–16)	8 (6–15)	0.372
Time to achieve full enteral feeding, days, median, (range)	8 (6–15)	8 (5–13)	0.232
Received BM/PDHM as initial enteral feeding, n (%)	19 (100)	18 (95)	0.311
Fed fortified BM/PDHM at chronological age 6 weeks, n (%)	19 (100)	15 (79)	0.105
Fortification with preterm formula, n (%)	16 (84)	17 (89)	0.631
Maximum total volume, mL/kg/day, mean ± SD	158 ± 10	160 ± 7	0.507
Age at which vitamin D started, day, median (range)	11 (8–15)	10 (8–14)	0.162

Abbreviations: BM, breast milk; PDHM, pasteurized donor human milk; PN, parenteral nutrition; IU, international units; SD, standard deviation. * *p* < 0.05 was considered statistically significant.

**Table 2:** Serum vitamin D level and metabolic bone laboratory parameters, and prevalence of hypovitaminosis D at pre- and post-vitamin D supplementation at age six weeks.

	Pre-Vitamin D Supplementation			Post-Vitamin D Supplementation		
	800 IU (n = 19)	400 IU (n = 19)	*p*-Value	800 IU (n = 19)	400 IU (n = 19)	*p*-Value
25(OH)D, ng/mL, mean ± SD	14.8 ± 4.8	14.7 ± 6.9	0.993	47.3 ± 21.0	32.0 ± 14.2	0.013 *
25(OH)D < 20 ng/mL, n (%)	17 (89)	14 (74)	0.209	1 (5)	6 (32)	0.036 *
25(OH)D < 12 ng/mL, n (%)	5 (26)	8 (42)	0.305	0 (0)	1 (5)	0.311
Serum calcium, mg/dL, mean ± SD	9.5 ± 0.7	9.9 ± 0.6	0.082	9.6 ± 0.5	9.8 ± 0.4	0.212
Serum phosphorus, mg/dL, mean ± SD	5.6 ± 1.4	5.8 ± 1.3	0.725	6.8 ± 1.7	6.6 ± 1.0	0.719
Serum alkaline phosphatase, U/L, mean ± SD	379 ± 238	308 ± 40	0.213	329 ± 106	327 ± 70	0.949
Serum albumin, g/dL, mean ± SD	3.3 ±0.5	3.3 ± 0.3	0.911	3.2 ± 0.5	3.4 ± 0.3	0.073

Abbreviations: 25(OH)D, 25-hydroxyvitamin D; ALP, alkaline phosphatase; SD, standard deviation; * *p* < 0.05 was considered statistically significant.

## Data Availability

The original contributions presented in this study are included in the article/Supplementary Material. Further inquiries can be directed to the corresponding author.

## Supplementary Materials

The following supporting information can be downloaded at: https://www.mdpi.com/article/10.3390/nu17111888/s1. The Supplementary Materials describe the nutritional protocols, fortification strategies, and vitamin D supplementation used in the study setting.
