# Supplementary material for: Improving Vitamin D Status in Preterm Newborns: A Randomized Trial of 800 vs. 400 IU/Day
Source: Nutrients. 2025 May 30;17(11):1888. doi: 10.3390/nu17111888 (PMC12158240; doi:10.3390/nu17111888)
Supplement: Supplementary file 1 [file nutrients-17-01888-s001.zip › nutrients-3649104-supplementary.pdf]

Supplementation protocol for preterm newborns at Maharaj Nakorn Chiang Mai Hospital

Criteria for receiving pasteurized donor human milk (PDHM)

- Preterm newborns with a birth weight of ≤1,800 g
- Newborns with surgical gastrointestinal diseases including gastroschisis, omphalocele, necrotizing enterocolitis, and short bowel syndrome

PDHM was discontinued when the infants' weight reached ≥1,800 g and/or when an adequate supply of maternal milk became available.

Fortification criteria

- Human milk fortifiers (HMF) or preterm formula were added when enteral feeding of ≥ 80 mL/kg/day, in accordance with hospital policy.
- HMF was the preferred option when available.

Routine nutritional supplementation for preterm newborns

At Maharaj Nakorn Chiang Mai Hospital, standardized nutritional protocols are applied to all preterm newborns with a birth weight of ≤ 1,500 g.

- Vitamin E: 25 IU/day
- Iron: 2-3 mg/kg/day
- Multivitamins: 0.5 mL/day
- Vitamin D supplementation: (total 600 IU/day)
    1) Additional vitamin D 400 IU/day
    2) Vitamin D3 from multivitamin 200 IU
- Mineral supplementation (if receiving <5 fortified feedings/day):
    1) Phosphorus: 60 mg/kg/day
    2) Calcium: 100 mg/kg/day

Forms of vitamin D supplementation in use

Vitamin D supplementation in Thailand is available in two main forms:

Isolated Vitamin D:

- Vitamin D2 (ergocalciferol): Commonly supplied in 20,000 IU capsules and locally prepared by dissolving in solution. At our hospital, pharmacists prepare it at a concentration of 1,000 IU/mL.

- Vitamin D3 (cholecalciferol): An oil-based isolated preparation (800IU/mL), preferred due to superior efficacy in raising serum 25(OH)D levels.

Multivitamins:

- Standard neonatal multivitamin formulations provide 200 IU of vitamin D3/0.5 mL.

Vitamin D supplementation in this study

- For this study, vitamin D3 was used as the primary form of supplementation.
- Target doses of either 400 IU/day or 800 IU/day were administered, depending on the newborn's group allocation.
- The supplementation was initiated when newborns achieved a feeding volume of at least 120 mL/kg/day.

Vitamin D in breast milk or pasteurized donor human milk with fortification

- *Human milk fortifiers* (Enfamil® Liquid Human Milk Fortifier, Mead Johnson, Zeeland, USA) contains vitamin D 188 IU/100 mL of 24 calories/oz concentration
- *Preterm formula* (ENFALAC A+ PREMATURE DHA+™, Mead Johnson, Netherlands) contains vitamin D 148 IU/100 mL of 24 calories/oz concentration
